# Activation of Propane C-H and C-C Bonds by Gas-Phase Pt Atom: A Theoretical Study

**DOI:** 10.3390/ijms13079278

**Published:** 2012-07-24

**Authors:** Fang-Ming Li, Hua-Qing Yang, Ting-Yong Ju, Xiang-Yuan Li, Chang-Wei Hu

**Affiliations:** 1 College of Chemical Engineering, Sichuan University, Chengdu, Sichuan, 610065, China; E-Mails: lifangming123@163.com (F.-M.L.); juty204@yahoo.cn (T.-Y.J.); xyli@scu.edu.cn (X.-Y.L.); 2 Key Laboratory of Green Chemistry and Technology, Ministry of Education, College of Chemistry, Sichuan University, Chengdu 610064, China; E-Mail: changweihu@scu.edu.cn

**Keywords:** Pt atom, propane, C-H bond, C-C bond, CCSD(T), BPW91

## Abstract

The reaction mechanism of the gas-phase Pt atom with C_3_H_8_ has been systematically investigated on the singlet and triplet potential energy surfaces at CCSD(T)//BPW91/6-311++G(d, p), Lanl2dz level. Pt atom prefers the attack of primary over secondary C-H bonds in propane. For the Pt + C_3_H_8_ reaction, the major and minor reaction channels lead to PtC_3_H_6_ + H_2_ and PtCH_2_ + C_2_H_6_, respectively, whereas the possibility to form products PtC_2_H_4_ + CH_4_ is so small that it can be neglected. The minimal energy reaction pathway for the formation of PtC_3_H_6_ + H_2_, involving one spin inversion, prefers to start at the triplet state and afterward proceed along the singlet state. The optimal C-C bond cleavages are assigned to C-H bond activation as the first step, followed by cleavage of a C-C bond. The C-H insertion intermediates are kinetically favored over the C-C insertion intermediates. From C-C to C-H oxidative insertion, the lowering of activation barrier is mainly caused by the more stabilizing transition state interaction Δ*E*^≠^_int_, which is the actual interaction energy between the deformed reactants in the transition state.

## 1. Introduction

In recent years, the dehydrogenation of lower alkanes has gained great importance in natural and petroleum gas utilization [1,2]. Propane is a cheap and easily available raw material as it is produced through a number of petrochemical processes, while the propylene market demand is rapidly increasing. Accordingly, the dehydrogenation of propane is an interesting alternative route to propylene production. In the catalytic cracking of alkanes and catalytic dehydrogenation of alkanes, platinum based catalysts have received much attention. Elucidation of the role of isolated platinum units in heterogeneous catalysis can be aided by gas-phase study, which can provide insight into the intrinsic properties and reactivities of discrete and well-characterized catalytic species [3].

In particular, the activation of C-H and C-C bonds of propane by transition metals (neutral, cationic, or clusters) in the gas phase has been an active area of research that provides fundamental information on catalytic reaction mechanisms, kinetics, and thermodynamics [4,5]. Of the first-row transition metal series, for the activation of propane, the early members (Sc^+^ [6], Ti^+^ [7], and V^+^ [8]) exhibit efficiency for the dehydrogenation of propane. Co^+^ cation favors H_2_ over CH_4_ [9,10], whereas Fe^+^ and Ni^+^ cations favor CH_4_ over H_2_ [9,11]. Cr^+^ cation does not show any efficiency for the activation of propane [12]. Of the second-row transition metal series, for Nb^+^ [13], Mo^+^ [12,14,15], and Rh^+^ cations [16], the dehydrogenation of propane is efficient and the dominant process at low energies, whereas products resulting from both C-H and C-C cleavage processes are observable at high energies. Rh atoms are also effective for the H_2_ elimination from ethane and larger alkanes under kinetics technique experiment [17]. For the reaction of Ag^+^ with propane, the dehydrogenation and formation of AgH^+^ + R products are not observed, whereas the C-C bond cleavage is the predominant process [18]. Of the actinide ions, for the activation of propane, Th^+^, Pa^+^ and U^+^ cations are efficient for the dehydrogenation of propane. For the other cations (Np^+^, Pu^+^, Am^+^, and Cm^+^), no reactions are observed experimentally [19]. For Th^2+^ and U^2+^, both C-H and C-C cleavage products are effectively observed [20]. Finally, for [(MgO)*_n_*]^+^ clusters, the higher reactivity with propane is not specific to [(MgO)_2_]^+^, but has been also observed for (MgO)^+^ [21,22].

Concerning the 5d-series transition metal Pt (neutral, cationic, or clusters), the reactions with linear alkanes have been extensively explored by means of diverse experimental and theoretical methods [23–29]. It is reported that the transition metal Pt (neutral, cationic, or clusters) are the efficient C-H insertion agents [23–29]. However, as far as we know, although a few investigations of C-H insertion processes have focused on the neutral Pt atom [23–27], the C-C insertion has hardly been investigated in the gas phase.

In the present study, a complete mechanism of neutral Pt atom with propane along with both the C-H and C-C bond activation processes is investigated, which is necessary to enable us to determine the crucial steps and to either block or enhance particular steps to steer the reaction in the desired direction. The goals of the present investigation are as follows: (a) to provide reliable structures and chemically accurate energetics of the reactants, intermediates, transition states (TSs), and products; (b) to elucidate the rate-determining step and the selectivity-controlling step; and (c) to gain a better understanding of the preference of reaction pathway. Particularly, to shed some light on the intrinsic reactivity of platinum atom toward the propane activation, the trends in reactivity and competition among the C-H and C-C bond cleavage mechanisms are analyzed using the activation strain model of chemical reactivity [30,31]. The potential energy profiles on the singlet and triplet states are investigated, because spin crossing is often involved in the transition metal-containing reactions [7,15,20].

## 2. Computational Details

All calculations were carried out with the Gaussian 03 program [32]. Full geometry optimizations were run to locate all of the stationary points and TSs on the singlet and triplet potential energy surfaces (PESs) for the reactions of Pt atom with C_3_H_8_, using the BPW91 [33,34] method with 6-311++G(d, p) basis set for the carbon and hydrogen atoms [35,36], and the Lanl2dz basis set and the corresponding effective core potential (ECP) for platinum [37], namely BPW91/6-311++G(d, p), Lanl2dz. Meantime, the stability of the density function theory (DFT) wavefunction was tested [38,39]. If an instability was found, the wavefunction was reoptimized with appropriate reduction in constraints, and the stability tests and reoptimizations were repeated until a stable wavefunction was found [38,39]. Harmonic frequency calculations were run to characterize stationary points and to take corrections of zero-point energy (ZPE) into account. The intrinsic reaction coordinate (IRC) method was performed to track minimum energy paths from transition structure to the corresponding minima [40,41]. The dominant occupancies of natural bond orbitals for some species have been analyzed with the help of the natural bond orbital (NBO) analysis [42,43]. To further determine electron correction energies, the single-point calculation of various species based on the optimized BPW91/6-311++G(d, p), Lanl2dz geometries were then refined using CCSD(T) [44] method with the same basis sets, namely CCSD(T)//BPW91/6-311++G(d, p), Lanl2dz. Unless otherwise mentioned, all energies are relative to the ground-state reactants [Pt(^3^D) + C_3_H_8_] at the CCSD(T)//BPW91/6-311++G(d, p), Lanl2dz level, including ZPE correction obtained at the BPW91/6-311++G(d, p), Lanl2dz level.

## 3. Results and Discussion

Considering the present system, the BPW91/6-311G++(d, p), Lanl2dz level is suitable to reproduce experimental values of geometrical parameters of H_2_, Pt_2_, and PtH diatomic molecules [45]. Furthermore, the single-point calculation of various species were then refined at the CCSD(T)//BPW91/6-311++G(d, p), Lanl2dz level. Thereupon, the present theoretical method of CCSD(T)//BPW91/6-311++G(d, p), Lanl2dz should be appropriate and reliable for the Pt + C_3_H_8_ systems.

In this work, we will mainly discuss the following reactions of Pt atom with C_3_H_8_:

(1) $$\text{Pt}+{\text{C}}_{3}{\text{H}}_{8}\to {\text{PtC}}_{3}{\text{H}}_{6}+{\text{H}}_{2}$$

(2) $$\text{Pt}+{\text{C}}_{3}{\text{H}}_{8}\to {\text{PtCH}}_{2}+{\text{C}}_{2}{\text{H}}_{6}$$

(3) $$\text{Pt}+{\text{C}}_{3}{\text{H}}_{8}\to {\text{PtC}}_{2}{\text{H}}_{4}+{\text{CH}}_{4}$$

The above three product channels were divided into five sections: (i) C-H bond activation: dehydrogenation, (ii) C-C bond activation: deethanization, (iii) C-C bond activation: demethanation, (iv) comparison of C-H with C-C bond activation, and (v) activation strain analysis of the direct C-H and C-C bond cleavage.

### 3.1. C-H Bond Activation: Dehydrogenation

**Scheme 1:**
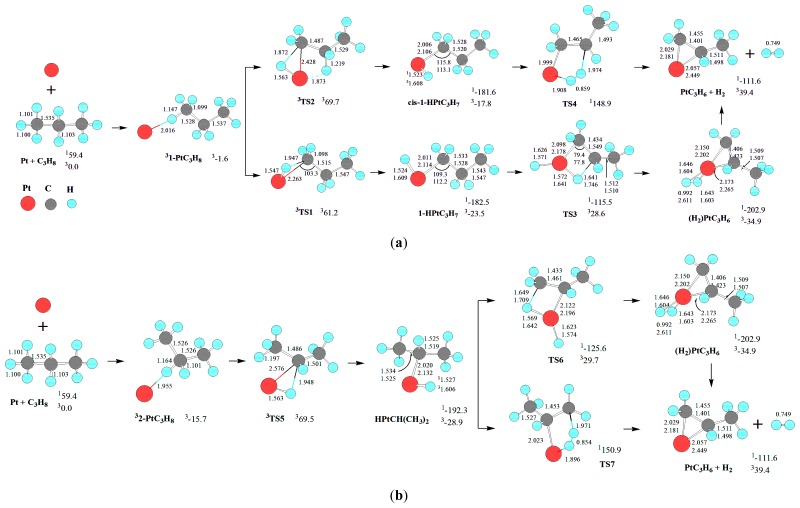

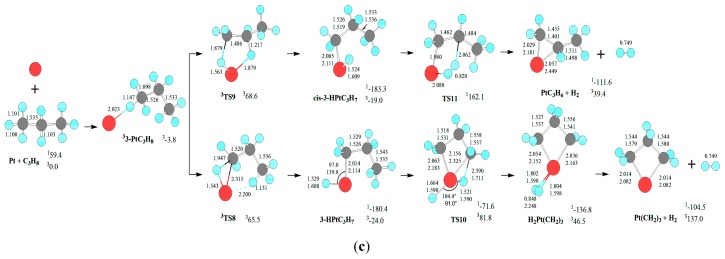
The reaction pathway and the optimized geometric structures of various species in the dehydrogenation of C_3_H_8_ by Pt atom, through (**a**) 1-PtC_3_H_8_; (**b**) 2-PtC_3_H_8_, and (**c**) 3-PtC_3_H_8_. Bond lengths are reported in Å and bonds angles in degree. Relative energies (kJ·mol^−1^) for the corresponding species relative to Pt(^3^D) + C_3_H_8_ at the CCSD(T)//BPW91/6-311++G(d, p), Lanl2dz level are shown.

For the dehydrogenation of C_3_H_8_ by Pt atom, the reaction pathway and the optimized geometric structures of various species are depicted in Scheme 1.

The triplet state Pt atom, ^3^D(d^9^s^1^), is the ground state. The singlet state Pt atom, ^1^S(d^10^), lies 59.4 kJ·mol^−1^ above the ground triplet state ^3^D(d^9^s^1^), in good agreement with the estimated value of 56.6 kJ·mol^−1^ [26]. The superscript prefixes “^1^” and “^3^” will be used to indicate the singlet and triplet states, respectively. As depicted in Scheme 1, there are two primary (*tans*-C-H^(1)^ and *cis*-C-H^(1)^ in the *trans* and *cis* position with respect to the CH_3_ group, respectively) and one secondary (C-H^(2)^) C-H bonds in propane. Then, with regard to the initial interaction between Pt atom and C_3_H_8_, three molecular complexes are considered: (i) Pt atom attacking the H-end of primary *tans*-C-H^(1)^ (1-PtC_3_H_8_), (ii) Pt atom attacking the H-end of secondary C-H^(2)^ approaching to Pt atom (2-PtC_3_H_8_), (iii) Pt atom attacking the H-end of primary *cis*-C-H^(1)^ (3-PtC_3_H_8_).

As discussed earlier, Pt atom has a triplet ground state (^3^D) with excitation energy of 59.4 kJ·mol^−1^ to the lowest singlet state (^1^S). Considering the initial interaction of Pt atom with C_3_H_8_, only the triplet ground state ^3^1-PtC_3_H_8_, ^3^2-PtC_3_H_8_, and ^3^3-PtC_3_H_8_ molecular complexes are obtained, whereas we failed to locate the corresponding ones on the singlet PES despite extensive attempts. For ^3^1-PtC_3_H_8_, ^3^2-PtC_3_H_8_, and ^3^3-PtC_3_H_8_, the BSSEs [46] by BPW91 are 10.1, 10.5, and 10.3 kJ·mol^−1^, and the complexation energies corrected by BSSEs are calculated to be 1.6, 15.7, and 3.8 kJ·mol^−1^ relative to the reactants Pt(^3^D) + C_3_H_8_, respectively. It is shown that the complex stability increases along ^3^1-PtC_3_H_8_ < ^3^3-PtC_3_H_8_ < ^3^2-PtC_3_H_8_. For ^3^1-PtC_3_H_8_, ^3^2-PtC_3_H_8_, and ^3^3-PtC_3_H_8_, the C-H bond close to Pt atom is elongated to 1.147, 1.164 and 1.147 Å from the 1.100, 1.103, and 1.101 Å of free C_3_H_8_, while there is a short Pt-H distance of 2.016, 1.955, 2.023 Å, respectively, indicating some molecular interaction between Pt atom and C_3_H_8_. The minimal energy reaction pathway (MERP) may start at the triplet molecular complexes (^3^1-PtC_3_H_8_, ^3^2-PtC_3_H_8_, and ^3^3-PtC_3_H_8_) from the corresponding ground triplet reactants.

As shown in Scheme 1, from these molecular complexes (1-PtC_3_H_8_, and 2-PtC_3_H_8_, 3-PtC_3_H_8_), the C-H bond cleavage may lead to the dehydrogenation product PtC_3_H_6_ (Pt-propene) + H_2_ and Pt(CH_2_)_3_ (Pt-cyclopropane) + H_2_. The change of Gibbs free energies (Δ*G*_298_) for the reactions of Pt(^3^D) + C_3_H_8_→^1^PtC_3_H_6_ (Pt-propene) + H_2_ and ^1^Pt(CH_2_)_3_ (Pt-cyclopropane) + H_2_ are calculated to be −111.6 and −104.5 kJ·mol^−1^, respectively. Thereby, the dehydrogenation of C_3_H_8_ is thermodynamically favorable.

For the formation of PtC_3_H_6_ (Pt-propene) + H_2_, there are five reaction pathways beginning at the three kinds of molecular complexes (two from 1-PtC_3_H_8_, two from 2-PtC_3_H_8_, and one from 3-PtC_3_H_8_), respectively, as shown in Scheme 1. Alternatively, for the formation of Pt(CH_2_)_3_ (Pt-cyclopropane) + H_2_, there is an unique reaction pathway starting from 3-PtC_3_H_8_.

First, from 1-PtC_3_H_8_, the initial primary C-H bond oxidative insertion via TS1 or TS2 leads to the σ-complex intermediate 1-HPtC_3_H_7_ or *cis*-1-HPtC_3_H_7_, respectively. From 1-HPtC_3_H_7_, a 1,2-dehydrogenation process takes place via four-center transition state TS3, resulting in a dihydrogen propene complex (H_2_)PtC_3_H_6_. Finally, the molecular complex (H_2_)PtC_3_H_6_ reductively eliminates H_2_, leaving PtC_3_H_6_ behind. Alternatively, from cis-1-HPtC_3_H_7_, a 1,2-dehydrogenation process occurs via five-center transition state TS4 directly, leading to the dissociation products PtC_3_H_6_ + H_2_.

Second, from 2-PtC_3_H_8_, the initial secondary C-H bond oxidative insertion via TS5 yields the σ-complex intermediate HPtCH(CH_3_)_2_. From HPtCH(CH_3_)_2_, there are two reaction pathways for the formation of PtC_3_H_6_ + H_2_. On the one hand, a 1,2-dehydrogenation process takes place via four-center transition state TS6, producing the dihydrogen propene complex (H_2_)PtC_3_H_6_. On the other hand, from HPtCH(CH_3_)_2_, a 1,2-dehydrogenation process occurs via five-center transition state TS7, directly resulting in the dissociation products PtC_3_H_6_ + H_2_.

Third, from 3-PtC_3_H_8_, the initial primary C-H bond oxidative insertion via TS8 or TS9 generates a σ-complex intermediate 3-HPtC_3_H_7_ or *cis*-3-HPtC_3_H_7_, respectively. From 3-HPtC_3_H_7_, a 1,3-dehydrogenation process takes place via five-member transition state TS10, generating the dihydrogen metallacycle molecular complex H_2_Pt(CH_2_)_3_. Last, the molecular complex dissociates into Pt(CH_2_)_3_ + H_2_. The structure of metallacycle Pt(CH_2_)_3_ is similar to that of Sc(CH_2_)_3_^+^ [6], TiC_3_H_6_^+^ [7], and NiC_4_H_8_^+^ [47]. Alternatively, from *cis*-3-HPtC_3_H_7_, a 1,2-dehydrogenation process occurs via five-center transition state TS11, producing the dihydrogen propene complex (H_2_)PtC_3_H_6_. Finally, (H_2_)PtC_3_H_6_ reductively eliminates H_2_, leaving PtC_3_H_6_ behind.

For the formation of PtC_3_H_6_ + H_2_, from 1-PtC_3_H_8_, the MERP should proceed via the minimal energy crossing point (MECP) between ^1^1-HPtC_3_H_7_ and ^3^1-HPtC_3_H_7_, with the energy height of the highest point (EHHP) of 61.2 kJ·mol^−1^ at ^3^TS1. From 2-PtC_3_H_8_, the MERP should proceed via MECP between ^1^HPtCH(CH_3_)_2_ and ^3^HPtCH(CH_3_)_2_, with the EHHP of 69.5 kJ·mol^−1^ at ^3^TS5. From 3-PtC_3_H_8_, the MERP should progress via MECP between ^1^*cis*-3-HPtC_3_H_7_ and ^3^*cis*-3-HPtC_3_H_7_, with the EHHP of 162.1 kJ·mol^−1^ at TS11. Since the EHHP at ^3^TS1 from 1-PtC_3_H_8_ is the lowest among the three reaction channels, this reaction channel for the formation of PtC_3_H_6_ + H_2_ is the most feasible kinetically. Furthermore, these results reveal a high preference of Pt atom for the attack of primary C-H bonds in propane, which is analogous to that of MgO^+^ cation for the attack of alkanes [21]. This feature represents a notable distinction of the transition-metal atom from various transition-metal oxide cations, which show a clear preference for the attack of secondary C-H bonds [21].

For the formation of Pt(CH_2_)_3_ + H_2_, from 3-PtC_3_H_8_, the MERP should advance via MECP between ^1^3-HPtC_3_H_7_ and ^3^3-HPtC_3_H_7_, with the EHHP of 65.5 kJ·mol^−1^ at ^3^TS8.

Moreover, the ^1^1-HPtC_3_H_7_, ^1^*cis*-1-HPtC_3_H_7_, ^1^HPtCH(CH_3_)_2_, ^1^3-HPtC_3_H_7_, ^1^*cis*-3-HPtC_3_H_7_, and ^1^(H_2_)PtC_3_H_6_ intermediates lie −182.5, −181.6, −192.3, −180.4, −183.3, and −202.9 kJ·mol^−1^ in a deep energetic well on each MERP, respectively. Then, these intermediates are thermodynamically favored in the dehydrogenation of C_3_H_8_. For the intermediates containing –PtH and –Pt-alkyl moieties (^1^1-HPtC_3_H_7_, ^1^*cis*-1-HPtC_3_H_7_, ^1^HPtCH(CH_3_)_2_, ^1^3-HPtC_3_H_7_, and ^1^*cis*-3-HPtC_3_H_7_), the NBO results show that a complete σ-bond has been formed both in Pt-H and in Pt-C.

From Pt + C_3_H_8_ to the C-H insertion intermediates (^1^1-HPtC_3_H_7_, ^1^*cis*-1-HPtC_3_H_7_, ^1^HPtCH(CH_3_)_2_, ^1^3-HPtC_3_H_7_, and ^1^*cis*-3-HPtC_3_H_7_), only the triplet molecular complexes and the triplet TSs are obtained, while we failed to gain the corresponding singlet ones, despite extensive attempts. Furthermore, for Pt atom (10 valence electrons), Pt(^1^S) singlet state, has an empty orbital and five doubly occupied nonbonding orbitals, whereas Pt(^3^D) triplet state has all of its s and d valence orbitals occupied, with four doubly occupied nonbonding orbitals, and two singly occupied nonbonding orbitals. That is to say, the bonding capacity of Pt(^1^S) singlet state to C_3_H_8_ is stronger than that of Pt(^3^D) triplet state. Then, the binding of Pt to C_3_H_8_ in the C-H insertion intermediates (1-HPtC_3_H_7_, *cis*-1-HPtC_3_H_7_, HPtCH(CH_3_)_2_, 3-HPtC_3_H_7_, and *cis*-3-HPtC_3_H_7_) inverts the energies of the singlet and triplet states from the Pt atom. Therefore, the ground state of the C-H insertion intermediates is the singlet state, as depicted in Scheme 1. The reaction goes forward from the excited state reactants Pt(^1^S) + C_3_H_8_ to the C-H inserted intermediates, without energy barrier. This can be ascribed to the fact that the singlet state Pt(^1^S) has an empty orbital, which should greatly facilitate the interaction with propane and its bond activation, leading smoothly to the formation of two covalent bonds to H and C_3_H_7_. This phenomenon has also appeared in the analogous Pt + CH_4_ system [26].

### 3.2. C-C Bond Activation: Deethanization

**Scheme 2:**
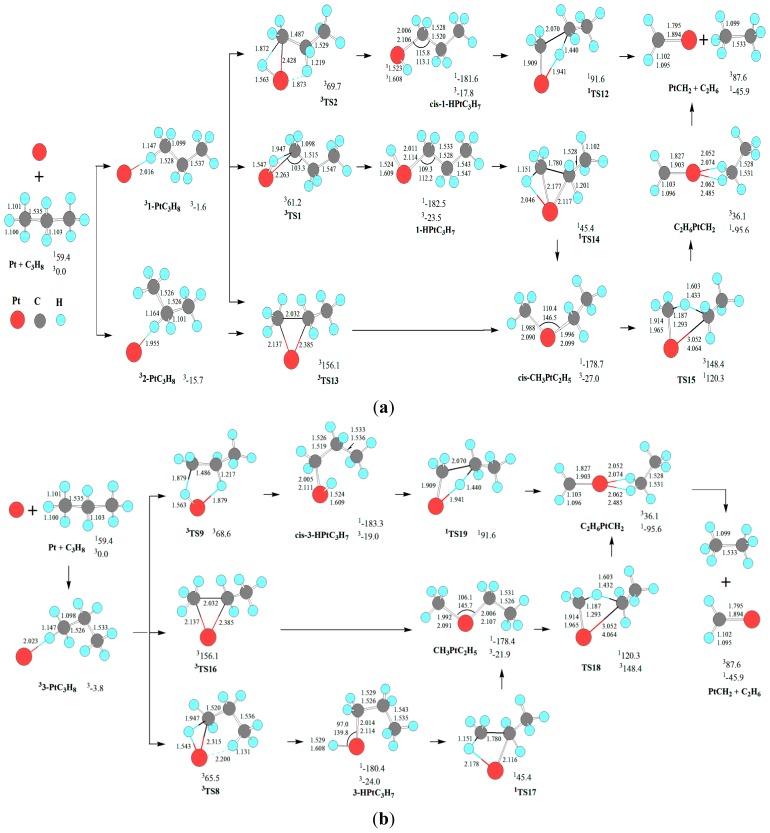
The reaction pathway and the optimized geometric structures of various species in the deethanization of C_3_H_8_ by Pt atom, through (**a**) 1-PtC_3_H_8_ and 2-PtC_3_H_8_, and (**b**) 3-PtC_3_H_8_. Bond lengths are reported in Å and bonds angles in degree. Relative energies (kJ·mol^−1^) for the corresponding species relative to Pt(^3^D) + C_3_H_8_ at the CCSD(T)//BPW91/6-311++G(d, p), Lanl2dz level are shown.

For the deethanization of C_3_H_8_ by Pt atom, the reaction pathway and the optimized geometric structures of various species are depicted in Scheme 2. The change of Gibbs free energies (Δ*G*_298_) for the reaction of Pt(^3^D) + C_3_H_8_→^1^PtCH_2_ + C_2_H_6_ is calculated to be −45.9 kJ·mol^−1^. Thereupon, the deethanization of C_3_H_8_ is thermodynamically favorable. Then, it is necessary to discuss kinetically the above reaction infra.

As shown in Scheme 2, for the deethanization of C_3_H_8_ by Pt atom, there are seven reaction pathways, three from 1-PtC_3_H_8_, one from 2-PtC_3_H_8_, and three from 3-PtC_3_H_8_. These seven reaction pathways are separated into two kinds of reaction pathways, one through the initial C-C bond direct cleavage, and another through the σ-complex assisted C-C σ-bond metathesis. For simplicity, we will primarily discuss the three reaction pathways from 1-PtC_3_H_8_ infra, which are analogous to those from 2-PtC_3_H_8_ and 3-PtC_3_H_8_. The nuances in energy mainly stem from their configuration differences among the three kinds of reaction channels.

As mentioned earlier, from 1-PtC_3_H_8_, there are three reaction pathways for the PtCH_2_ + C_2_H_6_ formation. For the first reaction pathway, Pt atom firstly inserts the C-H bond via five-member TS2, resulting in the intermediate *cis*-1-HPtC_3_H_7_. Then, from cis-1-HPtC_3_H_7_, a σ-complex assisted C-C σ-bond metathesis takes place via a four-member TS12 with both 1,3-H migration and C-C cleavage, yielding the molecular complex, C_2_H_6_PtCH_2_. Finally, C_2_H_6_PtCH_2_ releases C_2_H_6_ molecule, leaving PtCH_2_ behind. The MERP should proceed via the MECP between ^1^*cis*-1-HPtC_3_H_7_ and ^3^*cis*-1-HPtC_3_H_7_, with the highest energy requirement (HER) of 273.2 kJ·mol^−1^ at the ^1^*cis*-1-HPtC_3_H_7_→^1^TS12 reaction step and the EHHP of 91.6 kJ·mol^−1^ at ^1^TS12.

For the second and third reaction pathways from 1-PtC_3_H_8_, there are two reaction pathways to produce *cis*-CH_3_PtC_2_H_5_. That is, Pt atom directly inserts the C-C bond via three-member TS13, leading to the intermediate *cis*-CH_3_PtC_2_H_5_. Alternatively, Pt atom firstly inserts the C-H bond via the three-member TS1, resulting in the intermediate 1-HPtC_3_H_7_. Next, from 1-HPtC_3_H_7_, a σ-complex assisted C-C σ-bond metathesis occurs via a four-member TS14 with both 1,2-H shift and C-C bond cleavage, also yielding *cis*-CH_3_PtC_2_H_5_. Then, from CH_3_PtC_2_H_5_, the σ-complex assisted C-H σ-bond metathesis takes place via a four-member TS15 with 1,3-H shift, yielding the molecular complex C_2_H_6_PtCH_2_. As mentioned earlier, C_2_H_6_PtCH_2_ releases C_2_H_6_ molecule, staying PtCH_2_ behind. For the two reaction pathways, each MERP should advance via the MECP between ^1^*cis*-CH_3_PtC_2_H_5_ and ^3^*cis*-CH_3_PtC_2_H_5_, with the HER of 299.0 kJ·mol^−1^ at the ^1^*cis*-CH_3_PtC_2_H_5_→^1^TS15 reaction step. The two MERPs involve the EHHP of 156.1 and 120.3 kJ·mol^−1^ at ^3^TS13 and ^1^TS15, respectively.

Comparing these three reaction pathways, one can conclude that the reaction pathway via TS2 involving the first C-H cleavage and via TS12 involving C-C cleavage with synchronous 1,3-H migration is the gross MERP for the 1-PtC_3_H_8_→PtCH_2_ + C_2_H_6_ reaction, owing to its comparatively low HER (273.2 *vs.* 299.0 kJ·mol^−1^) and low EHHP (91.6 *vs.* 120.3 and 156.1 kJ·mol^−1^), with the rate-determining step of ^1^*cis*-1-HPtC_3_H_7_→^1^TS12→^1^C_2_H_6_PtCH_2_.

From 2-PtC_3_H_8_ for the formation of PtCH_2_ + C_2_H_6_, only one reaction pathway is obtained, which includes the initial C-C bond cleavage and σ-complex assisted C-H σ-bond metathesis. This reaction pathway is homologous to that via the initial C-C cleavage from 1-PtC_3_H_8_ with the HER of 299.0 kJ·mol^−1^ and the EHHP of 156.1 kJ·mol^−1^ at ^3^TS13.

From 3-PtC_3_H_8_ for the formation of PtCH_2_ + C_2_H_6_, there are also three reaction pathways. These three reaction pathways are similar to those from 1-PtC_3_H_8_. The reaction pathway of σ-complex assisted C-C σ-bond metathesis via ^3^TS9 and ^1^TS19 is kinetically most preferable in the three reaction pathways, because of its lowest HER (274.9 *vs.* 298.7 kJ·mol^−1^) and lowest EHHP (91.6 *vs.* 120.3 and 156.1 kJ·mol^−1^).

In summary, for the formation of the C-C cleavage products PtCH_2_ + C_2_H_6_, the optimal pathway proceeds through the σ-complex *cis*-1-HPtC_3_H_7_ or *cis*-3-HPtC_3_H_7_ from initial C-H bond cleavage, which assists the C-C σ-bond metathesis. This reactivity mode is also complementary for the classical reactivity picture through the direct C-C cleavage intermediate (M = Fe^+^ [48] and Ta^+^ [49]).

### 3.3. C-C Bond Activation: Demethanation

**Scheme 3:**
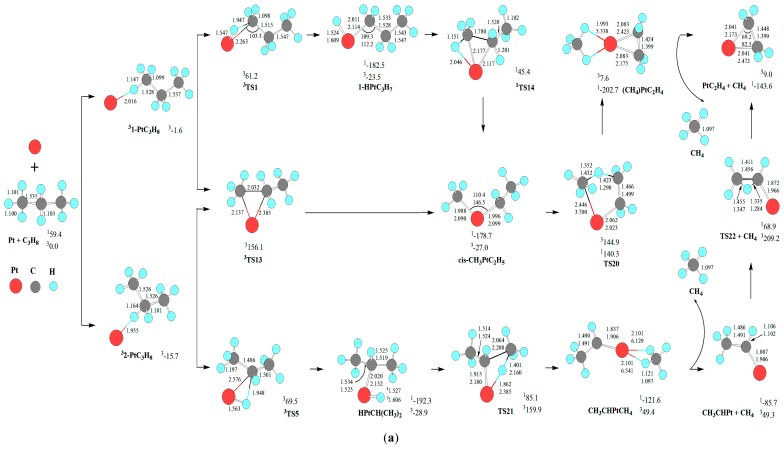

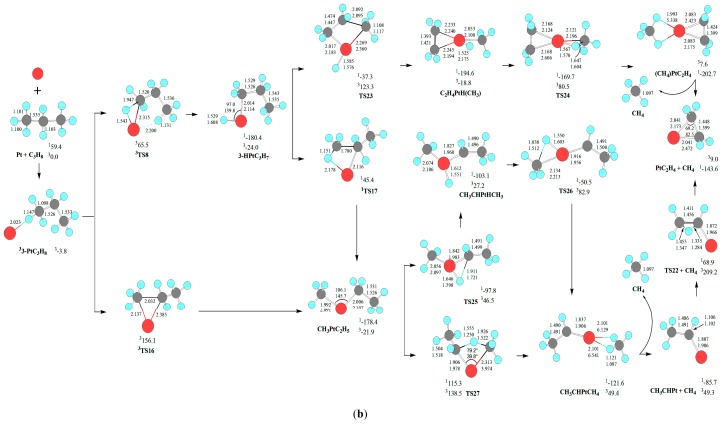
The reaction pathway and the optimized geometric structures of various species in the demethanation of C_3_H_8_ by Pt atom, through (**a**) 1-PtC_3_H_8_ and 2-PtC_3_H_8_, and (**b**) 3-PtC_3_H8. Bond lengths are reported in Å and bonds angles in degree. Relative energies (kJ·mol^−1^) for the corresponding species relative to Pt(^3^D) + C_2_H_6_ at the CCSD(T)//BPW91/6-311++G(d, p), Lanl2dz level are shown.

For the demethanation of C_3_H_8_ by Pt atom, the reaction pathway and the optimized geometric structures of various species are depicted in Scheme 3. The change of Gibbs free energies (Δ*G*_298_) for the reactions of Pt(3D) + C_3_H_8_→1PtC_2_H_4_ + CH_4_ are calculated to be −143.6 kJ·mol^−1^, which is thermodynamically favorable. Afterwards, we will discuss the kinetics of the above reaction infra. As shown in Scheme 3, there are four kinds of reaction pathways, which are through *cis*-CH_3_PtC_2_H_5_, CH_3_PtC_2_H_5_, HPtCH(CH_3_)_2_, and C_2_H_4_PtH(CH_3_) intermediates, respectively.

First, from *cis*-CH_3_PtC_2_H_5_, a σ-complex assisted C-H σ-bond metathesis takes place via a four-member TS20 with 1,4-H shift, yielding the molecular complex CH_4_PtC_2_H_4_. The molecular complex CH_4_PtC_2_H_4_ releases CH_4_ molecule, leaving PtC_2_H_4_ behind. Through *cis*-CH_3_PtC_2_H_5_, the MERP should go forward via the MECP between ^1^1-HPtC_3_H_7_ and ^3^1-HPtC_3_H_7_, with the HER of 319.0 kJ·mol^−1^ at the ^1^*cis*-CH_3_PtC_2_H_5_→^1^TS20 reaction step and the EHHP of 140.3 kJ·mol^−1^ at ^1^TS20.

Second, from CH_3_PtC_2_H_5_, a σ-complex assisted C-H σ-bond metathesis occurs via a four-member TS27 with 1,3-H shift, yielding a molecular complex CHCH_3_PtCH_4_. Then, the molecular complex CH_4_PtCHCH_3_ sets a CH_4_ molecule free, leaving PtCHCH_3_ behind. Next, from PtCHCH_3_, 1,2 H shift occurs via a four-member TS22, staying PtC_2_H_4_ behind. Through CH_3_PtC_2_H_5_, the MERP should go forward via the MECP between ^1^3-HPtC_3_H_7_ and ^3^3-HPtC_3_H_7_, with the HER of 225.8 kJ·mol^−1^ at the ^1^3-HPtC_3_H_7_→^1^TS17 reaction step and the EHHP of 68.9 kJ·mol^−1^ at ^1^TS22.

Third, from HPtCH(CH_3_)_2_, a σ-complex assisted C-C σ-bond metathesis occurs via a four-member TS21 with 1,3-H shift, also leading to the molecular complex CHCH_3_PtCH_4_. The MERP should go forward via the MECP between ^1^2-HPtC_3_H_7_ and ^3^2-HPtC_3_H_7_, with the HER of 277.4 kJ·mol^−1^ at the ^1^HPtCH(CH_3_)_2_→^1^TS21 reaction step and the EHHP of 85.1 kJ·mol^−1^ at ^1^TS21.

Fourth, from 3-HPtC_3_H_7_, an oxidative insertion of C-C bond to the platinum center takes place via a four-member TS23, producing a methyl hydrid complex C_2_H_4_PtH(CH_3_). Then, from C_2_H_4_PtH(CH_3_), 1,2-H shift occurs, yielding the molecular complex CH_4_PtC_2_H_4_. Last, the molecular complex CH_4_PtC_2_H_4_ sets a CH_4_ molecule free, leaving PtC_2_H_4_ behind. The MERP should go forward via the MECP between ^1^3-HPtC_3_H_7_ and ^3^3-HPtC_3_H_7_, with the HER of 143.1 kJ·mol^−1^ at the ^1^3-HPtC_3_H_7_→^1^TS23 reaction step and the EHHP of 65.5 kJ·mol^−1^ at ^3^TS8.

Comparing these four kinds of reaction pathways, one can see that the reaction pathway starting at the 3-PtC_3_H_8_ involving the crucial intermediate C_2_H_4_PtH(CH_3_) is the most optimal MERP for the Pt + C_3_H_8_→PtC_2_H_4_ + CH_4_ reaction, thanks to the lowest HER (143.1 *vs.* 319.0, 225.8, and 277.4 kJ·mol^−1^) and lowest EHHP (65.5 *vs.* 140.3, 85.1, and 68.9 kJ·mol^−1^), with the rate-determining step of ^1^3-HPtC_3_H_7_→^1^TS23→C_2_H_4_PtH(CH_3_). Thereby, the optimal pathway proceeds through the σ-complex 3-HPtC_3_H_7_ from initial C-H bond cleavage, which assists the C-C σ-bond metathesis. That is to say, the optimal C-C bond cleavages are assigned to C-H bond activation as the first step, followed by cleavage of a C-C bond. This reactivity mode is complementary for the classical reactivity picture through the direct C-C cleavage intermediate (M = Fe^+^ [48], and Ta^+^ [49]).

### 3.4. Comparison of C-H with C-C Bond Activation

As shown in Schemes 1–3, the C-H insertion intermediates (^1^1-HPtC_3_H_7_, ^1^*cis*-1-HPtC_3_H_7_, ^1^HPtCH(CH_3_)_2_, ^1^3-HPtC_3_H_7_, ^1^*cis*-3-HPtC_3_H_7_) and the C-C insertion intermediates (^1^CH_3_PtC_2_H_5_, ^1^*cis*-CH_3_PtC_2_H_5_, ^1^C_2_H_4_PtH(CH_3_), and ^1^CH_4_PtC_2_H_4_) deposit in a deep well, respectively. It is indicated that these intermediates are thermodynamically preferred. For the formation of the C-H and the C-C insertion intermediates, the corresponding MERP should involve the HER of about 60~70 and 140~230 kJ·mol^−1^, respectively. Thereby, the C-H insertion intermediates are kinetically favored, while the C-C insertion intermediates are kinetically hindered by energy barriers. These results are in qualitative agreement with the experimental results, in which the C-H insertion product is experimentally observed and the C-C insertion product is not formed in observable quantity in Pt + C_2_H_6_ system [27].

For the formation of C-C bond cleavage intermediates ^1^CH_3_PtC_2_H_5_ and ^1^*cis*-CH_3_PtC_2_H_5_, one can see that the reaction pathways of the direct C-C activation via ^3^TS16 and ^3^TS13 are inferior to those of the σ-complex assisted C-C σ-bond metathesis via ^1^TS17 and ^1^TS14 from 3-HPtC_3_H_7_ and 1-HPtC_3_H_7_, respectively, because of their higher EHHP (156.1 *vs.* 65.5 and 61.2 kJ·mol^−1^). This is reminiscent of the important role of σ-complex assistance for the C-C σ-bond metathesis. In other words, the direct C-C bond activation is associated with a sizable barrier, which would prohibit this channel.

A glance to the reaction pathways shown in Schemes 1–3 reveals that two kinds of σ-complexes (1-HPtC_3_H_7_, HPtCH(CH_3_)_2_, and 3-HPtC_3_H_7_) and (*cis*-1-HPtC_3_H_7_ and *cis*-3-HPtC_3_H_7_) from initial C-H bond cleavage are crucial for the selective formation of the final C-H and C-C cleavage products.

First, from the identical intermediate 1-HPtC_3_H_7_, the reaction step of ^1^1-HPtC_3_H_7_→^1^TS3→^1^(H_2_)PtC_3_H_6_ is competitive with that of ^1^1-HPtC_3_H_7_→^1^TS14→^1^*cis*-CH_3_PtC_2_H_5_. Because ^1^TS3 lies 160.9 kJ·mol^−1^ below ^1^TS14, (H_2_)PtC_3_H_6_ is selectively preferred, whereas *cis*-CH_3_PtC_2_H_5_ is selectively hampered. In other words, from 1-HPtC_3_H_7_, the dehydrogenation process dominates.

Second, from the identical intermediate *cis*-1-HPtC_3_H_7_, the reaction step of ^1^*cis*-1-HPtC_3_H_7_→^1^TS12→^1^C_2_H_6_PtCH_2_ is competitive with that of ^1^*cis*-1-HPtC_3_H_7_→^1^TS4→PtC_3_H_6_ + H_2_. Since ^1^TS12 locates 57.3 kJ·mol^−1^ below ^1^TS4, C_2_H_6_PtCH_2_ is selectively favored. That is to say, from *cis*-1-HPtC_3_H_7_, the deethanization process predominates.

Third, from the identical intermediate HPtCH(CH_3_)_2_, these reaction steps of HPtCH(CH_3_)_2_→^1^TS6→(H_2_)PtC_3_H_6_, HPtCH(CH_3_)_2_→^1^TS7→PtC_3_H_6_ + H_2_, and HPtCH(CH_3_)_2_→^1^TS21→CH_3_CHPtCH_4_ are competitive. As ^1^TS6 lies 276.5 and 210.7 kJ·mol^−1^ below ^1^TS7 and ^1^TS21, respectively, (H_2_)PtC_3_H_6_ is selectively preferred. Then, from HPtCH(CH_3_)_2_, the dehydrogenation process dominates.

Fourth, from the identical intermediate 3-HPtC_3_H_7_, these reaction steps of 3-HPtC_3_H_7_→^1^TS10→(H_2_)Pt(CH_2_)_3_, 3-HPtC_3_H_7_→^1^TS17→CH_3_PtC_2_H_5_, and 3-HPtC_3_H_7_→^1^TS23→C_2_H_4_PtH(CH_3_) are competitive. Because ^1^TS10 locates 117.0 and 34.3 kJ·mol^−1^ below ^1^TS17 and ^1^TS23, respectively, (H_2_)Pt(CH_2_)_3_ is selectively favored, whereas CH_3_PtC_2_H_5_ and C_2_H_4_PtH(CH_3_) are selectively hindered. Thereby, from 3-HPtC_3_H_7_, the dehydrogenation process dominates.

Last, from the identical intermediate *cis*-3-HPtC_3_H_7_, these reaction steps of *cis*-3-HPtC_3_H_7_→^1^TS11 →PtC_3_H_6_ + H_2_ and *cis*-3-HPtC_3_H_7_→^1^TS19→^1^C_2_H_6_PtCH_2_ are competitive. Because ^1^TS19 lies 70.5 kJ·mol^−1^ below ^1^TS11, ^1^C_2_H_6_PtCH_2_ is selectively preferred. Therefore, from *cis*-3-HPtC_3_H_7_, the deethanization process predominates.

In summary, once the σ-complex [1-HPtC_3_H_7_, or HPtCH(CH_3_)_2_, or 3-HPtC_3_H_7_] is formed, the major reaction channel results in the dehydrogenations products. Alternatively, as far as the σ-complex [*cis*-1-HPtC_3_H_7_ or *cis*-3-HPtC_3_H_7_] is concerned, the major reaction channel leads to the deethanization products. Besides, the demethanation process is kinetically ruled out.

To estimate quantitatively the reactivity and selectivity for the two kinds of products [PtC_3_H_6_ + H_2_ and PtCH_2_ + C_2_H_6_], the rate constants have been evaluated according to conventional transition state theory (TST) [50], including tunneling correction based on Winger’s formulation [51]. The formation of rate constant *k*(T) including tunneling correction coefficient *κ*(T) in transition state theory is given by

(4) $$k(\text{T})=k^{\prime }(\text{T})*\kappa (\text{T})$$

The rate constant *k*′(*T*) is simply given by

(5) $$k^{\prime }(\text{T})=\frac{k_{B}T}{hc^{0}}e^{\frac{-\Delta G^{*}}{RT}}$$

where *k*_B_ is the Bolzmann constant, *h* is the Planck constant, *T* is thermodynamic temperature, *c*^0^ is standard concentration, and Δ*G*^≠^ is Gibbs free energy. The tunneling correction coefficient *κ*(T) is written in the form of

(6) $$\kappa (\text{T})=1+\frac{1}{24}{\left|\frac{h\nu^{\neq }}{k_{B}T}\right|}^{2}$$

where *k*_B_ is the Bolzmann constant, *h* is the Planck constant, *T* is thermodynamic temperature, and *v*^≠^ is the imaginary frequency of the unbound normal mode at the saddle point. The branching ratio (*α*_i_) of product *i* is calculated by

(7) $$\alpha_{\text{i}}=\frac{k_{i}(\text{T})}{\Sigma k_{i}(\text{T})}$$

where *k*_i_ is the rate constant of product *i*.

From the identical reactants Pt + C_3_H_8_, the formation of PtC_3_H_6_ + H_2_ and PtCH_2_ + C_2_H_6_ are competitive, while their selectivity-controlling steps are Pt(^3^D) + C_3_H_8_→^3^TS1→^3^1-HPtC_3_H_7_ and Pt(^3^D) + C_3_H_8_ → ^3^TS9→^3^*cis*-3-HPtC_3_H_7_ on their MERPs, respectively. Thereby, the rate constants were taken into account, where Pt(^3^D) + C_3_H_8_ were taken as reactants, while ^3^TS1 and ^3^TS9 served as TSs, respectively. The rate constants for the formation of ^3^1-HPtC_3_H_7_ (*k*_1_) and ^3^*cis*-3-HPtC_3_H_7_ (*k*_2_) calculated over 300–1100 K temperature range can be fitted by the following expressions (in dm^3^·mol^−1^·s^−1^):

(8) $$k_{1}=6.65\times 10^{8}\;\text{exp}(-65,855/RT)$$

(9) $$k_{2}=2.85\times 10^{8}\;\text{exp}(-72,999/RT)$$

The branching ratios for the formation of PtC_3_H_6_ + H_2_ and PtCH_2_ + C_2_H_6_ are calculated to be 97.7~83.6% and 2.3~16.4%, respectively, over 300–1100 K temperature range. In other words, the dehydrogenation channel is predominant, and the deethanization channel is minor, while the demethanation channel is ruled out.

### 3.5. Activation Strain Analysis of the direct C-H and C-C Bond Cleavage

To gain insight into how the Pt atom affects the activation barriers of the initial C-H and C-C bond cleavage, *i.e.*, insight into how this effect depends on the nature of concomitant geometrical deformation and electronic structures of Pt and C_3_H_8_, the trends in reactivity and competition among the initial C-H and C-C bond mechanisms are analyzed using the activation strain model of chemical reactivity [30,31]. In this model, activation energies Δ*E*^≠^ of the TS are divided into the activation strain Δ*E*^≠^_strain_ and the stabilizing TS interaction Δ*E*^≠^_int_: Δ*E*^≠^ = Δ*E*^≠^_strain_ + Δ*E*^≠^_int_. The activation strain Δ*E*^≠^_strain_ is the strain energy associated with deforming the reactants from their equilibrium geometry to the geometry they adopt in the TS. The TS interaction Δ*E*^≠^_int_ is the actual interaction energy between the deformed reactants in the TS [30,31].

The results of the activation strain analysis are listed in Table 1. The activation energy Δ*E*^≠^ increases from the initial C–H cleavage TSs (^3^TS1, ^3^TS2, ^3^TS5, ^3^TS9, and ^3^TS8) of 60~70 kJ·mol^−1^ to the initial C–C cleavage TSs (^3^TS13 and ^3^TS16) of ~160 kJ·mol^−1^. The activation strain Δ*E*^≠^_strain_ decreases from the intial C–H cleavage TSs (^3^TS1, ^3^TS2, ^3^TS5, ^3^TS9, and ^3^TS8) of 290~300 kJ·mol^−1^ to the initial C–C cleavage TSs (^3^TS13 and ^3^TS16) of ~200 kJ·mol^−1^. The activation strain appears to be related to the bond strength of the activated bond and with the percentage-wise extent of bond stretching in the TS. Typical strengths and lengths of the C-H and C-C bonds are the following: 414 (C-H) and 347 kJ·mol^−1^ (C-C), and ~1.1 (C-H) and ~1.5 Å (C-C) [52]. Moreover, we recall that the percentage-wise extent of bond stretching in the TS for oxidative insertion is 70~80% (C-H) and ~30% (C-C). Hence, both the bond strength and the percentage-wise bond elongation in the TS decreases from C-H to C-C. This correlates nearly with the activation strain Δ*E*^≠^_strain_, which decreases in the same order. It is indicated that the activation strain prefers the C-C oxidative insertion over the C-H oxidative insertion. That is to say, the activation strain Δ*E*^≠^_strain_ makes in the reverse order as the activation energy Δ*E*^≠^, for the C–H and C–C oxidative insertion. Alternatively, the strength of the TS interaction increases from ~−50 kJ·mol^−1^ (C-C) to −220~−240 kJ·mol^−1^ (C-H). From C-C to C-H bond activation, the strengthening of the TS interaction varies among 170–190 kJ·mol^−1^, whereas the activation strain changes only by ~90 kJ·mol^−1^. Thereby, the lowering activation energy from C-C to C-H bond activation stems mainly from the strengthening of the TS interaction.

Thus, the stabilizing interaction Δ*E*^≠^_int_ prefers C-H oxidative insertion, whereas the activation strain Δ*E*^≠^_strain_ favors C-C oxidative insertion. From C-C to C-H oxidative insertion, the lowering of activation barrier is mainly caused by the TS interaction Δ*E*^≠^_int_ becoming more stabilizing.

## 4. Conclusions

The reaction mechanism of the gas-phase Pt atom with C_3_H_8_ has been systematically investigated on the singlet and triplet potential energy surfaces. Considering the initial interaction of Pt atom with C_3_H_8_, Pt atom prefers the attack of primary over secondary C-H bonds in propane. The major and minor reaction channels lead to the dehydrogenation products PtC_3_H_6_ + H_2_ and the deethanization products PtCH_2_ + C_2_H_6_, respectively, whereas the possibility to form the demethanation products PtC_2_H_4_ + CH_4_ is so small it can be neglected. Over the 300–1100 K temperature range, the branching ratios for the formation of PtC_3_H_6_ + H_2_ and PtCH_2_ + C_2_H_6_ are calculated to be 97.7%~83.6% and 2.3~16.4%, respectively. The MERP for the formation of the main products PtC_3_H_6_ + H_2_, involving one spin inversion, prefers to start at the triplet state and afterward proceed along the singlet state. The optimal C-C bond cleavages are assigned to C-H bond activation as the first step, followed by cleavage of a C-C bond. This reactivity mode is complementary for the classical reactivity picture through the direct C-C cleavage intermediate.

Furthermore, both the C-H insertion intermediates [1-HPtC_3_H_7_, *cis*-1-HPtC_3_H_7_, HPtCH(CH_3_)_2_, 3-HPtC_3_H_7_, *cis*-3-HPtC_3_H_7_] and the C-C insertion intermediates [CH_3_PtC_2_H_5_, *cis*-CH_3_PtC_2_H_5_, C_2_H_4_Pt(H)(CH_3_), and CH_4_PtC_2_H_4_] are thermodynamically preferred. However, the C-H insertion intermediates are kinetically favored over the C-C insertion intermediates. These results are in qualitative agreement with the experimental results, in which the C-H insertion product is experimentally observed and the C-C insertion product is not formed in observable quantity in Pt + C_2_H_6_ system.

Unlike the role of the activation strain Δ*E*^≠^_strain_, the stabilizing interaction Δ*E*^≠^_int_ favors the initial C-H oxidative insertion over the initial C-C oxidative insertion. From C-C to C-H oxidative insertion, the lowering of activation barrier is mainly caused by the TS interaction Δ*E*^≠^_int_ becoming more stabilizing.

## Figures and Tables

**Table 1:** Geometry (in Å) at the BPW91/6-311++G(d, p), Lanl2dz level and activation strain analysis of transition state (in kJ·mol^−1^) at the CCSD(T)//BPW91/6-311++G(d, p), Lanl2dz level for the C-H and C-C bond cleavage in C_3_H_8_ by Pt atom.

Activated bond	TS	Length in C_3_H_8_	Length in TS	Stretching in TS	Stretching in TS (in %)	Δ*E*^≠^_strain_	Δ*E*^≠^_int_	Δ*E*^≠^
C-H	^3^TS1	1.100	1.947	0.847	77.0	301.2	−240.0	61.2
C-H	^3^TS2	1.100	1.872	0.772	70.2	289.6	−219.9	69.7
C-H	^3^TS5	1.103	1.948	0.845	76.6	293.3	−223.8	69.5
C-H	^3^TS8	1.101	1.879	0.778	70.7	303.8	−238.3	65.5
C-H	^3^TS9	1.101	1.948	0.847	76.9	290.1	−221.5	68.6
C-C	^3^TS13	1.535	2.032	0.497	32.4	207.5	−51.4	156.1
C-C	^3^TS16	1.535	2.032	0.497	32.4	207.5	−51.4	156.1

## Appendix

Zero-point energies (*ZPE*) (hartree), total energies (*E*_c_) (hartree) corrected by ZPE, relative energies (*E*_r_) (kJ·mol^−1^) of various species at BPW91/6-311++G(d, p), Lanl2dz and CCSD(T)//BPW91/6-311++G(d, p), Lanl2dz levels with respect to the ground reactants Pt(^3^D) + C_3_H_8_. The standard orientations and vibrational frequencies of various species calculated at the BPW91/6-311++G(d, p), Lanl2dz level in the reactions of Pt + C_3_H_8_. The schematic energy diagrams in the activation of C_3_H_8_ by Pt atom calculated at the CCSD(T)//BPW91/6-311++G(d, p), Lanl2dz level, in which the relative energies (kJ·mol^−1^) for the corresponding species relative to Pt(^3^D) + C_3_H_8_ are shown.
